# Outdoor Pollution Comparison Between Bucharest and Its Outskirts Using Mobile Laboratory

**DOI:** 10.3390/ijerph21121573

**Published:** 2024-11-26

**Authors:** Razvan Stefan Popescu, Lelia Letitia Popescu, Tiberiu Catalina

**Affiliations:** 1 Buildings’ Services Faculty, Technical University of Civil Engineering of Bucharest, 020396 Bucharest, Romania; razvan.popescu@utcb.ro (R.S.P.); tiberiu.catalina@utcb.ro (T.C.); 2 National Research and Development Institute URBAN-INCERC, 021652 Bucharest, Romania

**Keywords:** outdoor pollution, mobile laboratory, PM_2.5_, PM_10_, BTEX

## Abstract

This study presents a modern mobile laboratory to monitor outdoor air quality in Bucharest, Romania, with a focus on pollutants associated with transportation. Particulate matter (PM_2_._5_, PM_10_), carbon monoxide (CO), ozone (O_3_), sulfur dioxide (SO_2_), nitrogen oxides (NO, NO_2_), and BTEX compounds (benzene, toluene, ethylbenzene, and xylenes) were among the significant pollutants that were examined in the lab. Meteorological variables such wind direction and speed, temperature, humidity, and solar radiation were also routinely observed in order to assess their influence on pollution levels. The study looked at two locations—a bustling city road in Bucharest and a remote community 40 kmawayin Snagov—under a range of weather conditions, including sunny, rainy, warm, and chilly days. The findings showed that the primary source of pollution in the urban area, which had significantly higher pollution levels than the rural site, was transportation. Particularly in the city, alarming concentrations of harmful particulate matter and carcinogens like benzene were found, underscoring the need for continuous air quality monitoring. The weather has a major impact on the dispersal of contaminants. Because of washout effects, rainy days decreased airborne pollutants, but sunny days showed higher pollution deposition. This study highlights the importance of outdoor air quality monitoring, particularly in urban environments, where traffic and weather have a significant impact on pollution levels. These findings provide crucial data that policymakers can utilize to implement targeted pollution control measures that protect human health.

## 1. Introduction

This study uses a contemporary mobile laboratory to analyze pollution caused by transportation. Carbon monoxide (CO), nitrogen oxides (NOx), sulfur dioxide (SO_2_), particulate matter (PM_2_._5_ and PM_10_), and BTEX compounds (benzene, toluene, ethylbenzene, o-, m-, and p-xylenes) are among the main pollutants released by internal combustion vehicles. As pollution sources intensify due to climate change, these emissions are predicted to rise in metropolitan areas [1].

Air pollution surpassed other chronic illness risk factors such as obesity, high cholesterol, and malnutrition to become the fourth most important worldwide risk factor for death in 2019 [2]. Additionally, this year was the first time that studies looked at the substantial effects of air pollution on infants, establishing a relationship between exposure to air pollution and 20% of newborn mortality globally. Premature births and low birth weight are linked to a large number of these deaths, which account for about 1.8 million deaths annually worldwide [3]. 

Particulate matter and gaseous pollutants combine to form a complex mixture of contaminants that make up outdoor air pollution [4]. It is now widely acknowledged that volatile organic compounds (VOCs), particularly those found in urban air pollution, have a significant negative influence on human health and contribute to the formation of photochemical oxidants such as ozone [5]. Although there are both biogenic and anthropogenic sources of VOCs, human-made sources—such as automobile exhaust emissions, gas station leaks, tobacco smoke, garbage decomposition, and industrial processes—are the primary contributors in urban areas [6]. Compared to anthropogenic sources, biogenic sources—like forests or wetlands—have less of an impact on urban air pollution [7].

Because of their link to automobile emissions and industrial processes, BTEX (benzene, toluene, ethylbenzene, and xylene) compounds account for a sizable amount of volatile organic compounds (VOCs) in urban settings. For instance, it has been claimed that the percentage of BTEX in total VOCs might approach 60% in Hong Kong [8], and could amount to 70% of ambient VOCs in Beijing during the summer [9] and around 33% in Dinghu Mountain and Beijing combined [10]. Of these substances, benzene is particularly dangerous and is categorized by the U.S. Environmental Protection Agency (EPA) as a Class A carcinogen and by the International Agency for Research on Cancer (IARC) as a Group 1 human carcinogen [11]. Long-term exposure to toluene vapor has also been demonstrated to have detrimental effects on the myelin of the central nervous system [12].

These chemicals’ effects on air quality are influenced by the wide variations in their atmospheric lifetimes. For instance, xylenes only last in the atmosphere for around 7.8 h, whereas benzene has an atmospheric lifetime of roughly 12.5 days, which is roughly six times longer than that of toluene [13]. The photo-oxidation rates of BTEX compounds also vary: toluene and m-xylene react five and nineteen times faster with OH radicals, respectively, than benzene, which contributes differentially to the potential for ozone generation [14]. Monitoring BTEX compound concentrations in indoor and outdoor contexts is still crucial for public health because of the health concerns and their role in ozone production [15].

BTEX (benzene, toluene, ethylbenzene, and xylenes) chemicals account for a large portion of total volatile organic compounds (VOCs) in metropolitan areas. BTEX, for example, accounts for over 60% of all VOCs in Hong Kong [16] and roughly 70% of ambient VOCs in Beijing during the summer [17]. BTEX makes up about 33% of all VOCs in Dinghu Mountain and some areas of Beijing, which is indicative of the significant impact of industrial activity and vehicle emissions [15,18].

The International Agency for Research on Cancer (IARC) has designated benzene as a Group 1 human carcinogen, and the U.S. Environmental Protection Agency (EPA) has designated it as a Class A carcinogen, making it the most dangerous of the BTEX chemicals [19]. The myelin of the central nervous system has been demonstrated to be negatively impacted by prolonged exposure to toluene fumes, highlighting the health hazards associated with BTEX chemicals [20].

These chemicals have different atmospheric lives; benzene lasts about 12.5 days, which is 6.25 times longer than toluene, whilst xylenes only last about 7.8 h. Furthermore, the rates of photo-oxidation for BTEX chemicals vary; toluene and m-xylene react five and nineteen times faster with hydroxyl (OH) radicals, respectively, than benzene, which contributes significantly to the generation of ozone and air quality issues [7].

In order to safeguard public health, it is crucial to keep an eye on the levels of BTEX compounds in both indoor and outdoor environments due to their significant health concerns and tendency to produce ozone [21]. In order to evaluate the health effects of prolonged exposure, researchers looked at BTEX levels in various environments as part of an environmental air quality study [22]. The high prevalence of BTEX chemicals in traffic-heavy locations was observed in another urban air pollution analysis [12]. Research has repeatedly demonstrated that these VOCs are associated with increased health hazards, especially in urban regions, where vehicle emissions predominate [23]. Regular monitoring is necessary, since the presence of BTEX in ambient air is linked to neurological and respiratory problems [24]. The significance of VOC assessments in urban planning and health policy has been emphasized by additional studies [25]. According to recent research, monitoring BTEX levels continuously can help lessen the negative impacts of air pollution [26]. Last but not least, research from a variety of sources indicates that proactive BTEX pollution management and monitoring are essential for public health [27].

In the urban region of Bandar Abbas, Iran, H.R. Ghaffari et al. recorded values of 14.60 ± 9.76 µg/m^3^ for toluene and 2.51 ± 2.06 µg/m^3^ for benzene. With a T/B ratio greater than 1, their investigation likewise revealed positive correlations between BTEX chemicals and air temperature, indicating that traffic-related sources are the main culprits [19].

Similarly, in Yazd, Iran, Y. Hajizadeh et al. found that the BTEX ratios were 1.5:2.7:1:2.9, with xylene being the main volatile organic compound (VOC), with an average concentration of 41 ± 45 µg/m^3^. The study found that higher levels of atmospheric precipitation were associated with a seasonal trend in benzene levels, which decreased from summer to winter [23].

According to da Silva et al., the average BTEX concentrations in the Bangu neighborhood of Rio de Janeiro, Brazil, were 1.5 µg/m^3^ for benzene, 6.7 µg/m^3^ for toluene, 1.5 µg/m^3^ for ethylbenzene, and 4.2 µg/m^3^ for xylenes [14]. Additionally, Bauri et al. discovered that toluene and xylenes were the main sources of BTEX levels in the urban atmosphere in Dehradun, India, underscoring the geographical diversity in VOC profiles [13].

Shi et al. reported that northern China’s PM_2_._5_ and NO_2_ levels decreased by about 29 ± 22% and 53 ± 10%, respectively, in response to lower economic activity during the COVID-19 outbreak in early 2020. Meanwhile, ozone concentrations rose by a factor of 2.0 ± 0.7, suggesting changes in pollution dynamics brought on by lower industrial and vehicle emissions [28].

For comparison, this study provides global BTEX concentration data in Table 1. Our study uses a mobile laboratory to quantify the air pollution caused by traffic in Bucharest, the capital of Romania, in 2021. This lab continuously tracks meteorological variables and chemical parameters (BTEX, Ozone, NOx, NO_2_, CO, SO_2_, PM_10_, and PM_2_._5_) to provide useful information that can direct local government initiatives to improve outdoor air quality and lower the related health risks.

## 2. Materials and Methods

**Figure 1:**
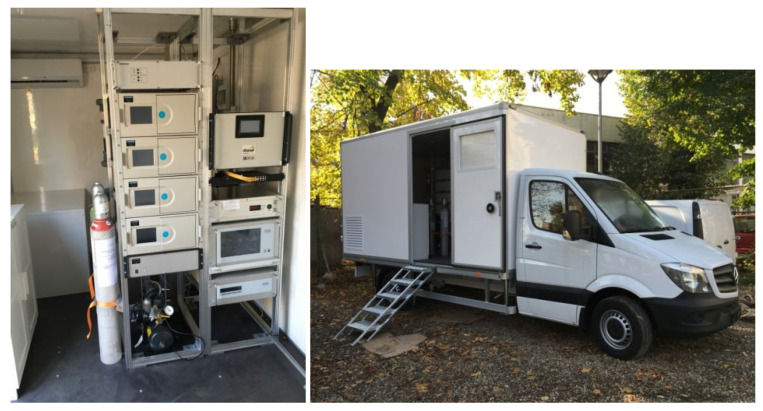
Mobile lab used for pollution measurements.

Our research employed an innovative mobile laboratory specifically engineered for pollution monitoring. Outdoor VOC levels are generally quantified by concentrating the chemicals on adsorbent materials, followed by thermal desorption and then measurement using gas chromatography (GC). This method is commonly employed for continuous outdoor pollution monitoring; however, the primary drawback is the high cost of the equipment. Figure 1 displays photos of the mobile laboratory utilized for these measurements. The laboratory is equipped to monitor BTEX compounds (benzene, toluene, ethylbenzene, and o-, m-, p-xylene), typically emitted by gasoline and diesel engines, alongside inorganic pollutants (SO_2_, CO, NOx, ozone), particulate matter (PM_2_._5_ and PM_10_), and meteorological parameters (wind speed and direction, temperature, humidity, pressure, precipitation, and solar radiation), irrespective of external weather conditions.

BTEX measurements are conducted via gas chromatography with a flame ionization detector (FID), which, despite its expense, yields accurate outdoor VOC values. The gas chromatograph features a 60 m capillary column and functions under the followingtemperature protocol: it is initially set at 50 °C for 3 min, then increases at a rate of 8 °C/min until it attains 180 °C, which is sustained for 5 min, culminating in a total duration of 18 min. The mobile laboratory is outfitted with an electric generator to facilitate remote measurements in areas without electrical grid access. Before each measurement campaign, all instruments are calibrated to guarantee data accuracy. Table 2 delineates the principal characteristics of the instruments employed for pollution monitoring.

Two dust monitors were utilized for particulate matter measurements: a GRIMM brand(Karlsruhe, Germany), Portable Environmental Dust Monitor model 11-E, which employs laser radiation to quantify PM_2_._5_, and a Verewa brand (Germany), monitor that utilizes beta-radiation for PM_10_ quantification. As the mobile laboratory was not initially designed to simultaneously monitor both PM_2_._5_ and PM_10_, an additional device was incorporated expressly for particulate matter assessment. The IOVIS software (https://iovis.com.tr/) installed in the system facilitates remote access to the collected data at any time.

## 3. Findings and Analysis

This study correlates the measured pollution levels with adjacent vehicular traffic and, where relevant, industrial operations. Two locations were examined (data collected in the year 2021):

- The student campus and the Faculty of the Technical University of Civil Engineering in Bucharest, situated on Pache Protopopescu Boulevard, a thoroughfare characterized by significant congestion during peak hours (designated as PP in this study);
- Snagov, a secluded town located 40 km from Bucharest, encircled by a forest and a lake, renowned as a weekend retreat for leisure and recreational pursuits.

**Figure 2:**
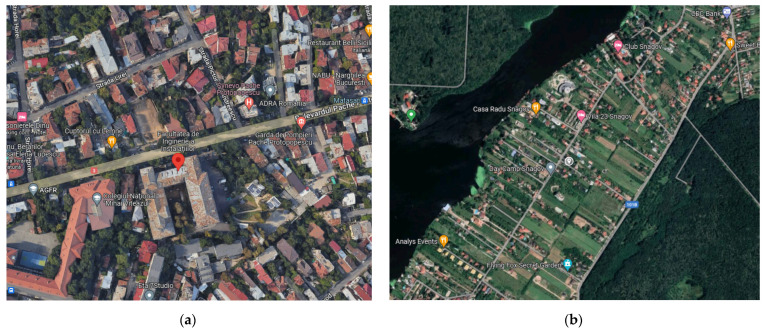
Measured locations, Bucharest (**a**) and Snagov (**b**).

**Figure 3:**
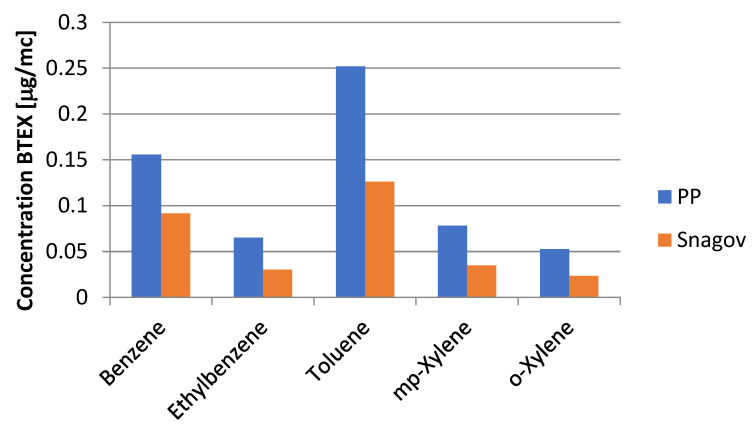
Daily mean BTEX concentrations between the two locations.

The two test sites (Figure 2)were chosen so that a comparison could be made between a clean, remote area like Snagov and a busy, urban area like Bucharest’s student campus. Figure 3 shows the average concentrations of BTEX compounds and compares the amounts of pollution. For example, the amount of benzene in the air in Snagov (9 November 2021) is almost half of what it is in Bucharest (25 August 2021). This is mostly because of the pollution in the city and the traffic on nearby roads. This study was conducted within the constraints of a four-month funding period, which limited the scope of data collection. As a result, we prioritized exploring the urban–rural differences during periods that were most representative of each region’s pollution dynamics (e.g., high-traffic vs. low-traffic seasons). While this was beyond the scope of the current study due to funding constraints, we view this as an important avenue for future research. As part of our ongoing efforts, we aim to expand this work to include multi-seasonal data collection in both environments (or more than two monitored places) to further enrich our findings.

Figure 3 also shows the types of toxins in the BTEX and how much of them is present on an average daily basis.

When you look at the BTEX levels in Snagov and the Pache Protopopescu campus (PP), you can see that there are big changes in the percentages of each compound and how much each one contributes to the total BTEX levels. Toluene makes up about 30% of the total BTEX in Snagov but almost 40% in PP. This is because there is more traffic and more pollution in the cities. Benzene, a major pollutant, makes up about 25% of all BTEX in Snagov but 35% in PP, which shows that pollution from vehicles has become a lot worse. Ethylbenzene and xylenes follow the same pattern, with 5–10% less of them in the total BTEX in Snagov than in PP. These percentages show not only that the overall concentrations are lower in Snagov, but also that the composition of BTEX pollutants has changed. This shows how strongly traffic affects air quality in places with lots of people.

Brocco et al. [38] found that traffic is the main source of pollution when the T/B ratio is greater than 1. Hsieh [39] et al. looked at how pollution sources at different distances affected the m,p X/E ratio and found that when the distance is very short, it goes above 3.3, which does not happen in our data. Khoder’s study [30], which compared BTEX ratios in different places around the world, showed that these ratios can help figure out whether pollution comes from factories or cars. The BTEX rates for PP and Snagov (Table 3) give us important information about the places where pollution comes from. In PP, the B/T ratio is 0.62, which is less than 1, which means that pollution comes from a number of different sources. However, traffic is expected to play a big role, as shown by the high T/E ratio (3.86). This backs up the conclusion that traffic is the main source when T/B is greater than 1. The B:T:E:X ratio at PP is 2.3:3.8:1:2. This is very similar to the traffic-related ratio of 3:4:1:5 that Khoder [30] found, which supports the idea that car emissions are a major cause at this site. The B/T ratio in Snagov is a little higher at 0.73, which shows that the pollution profile is different from that in PP. The low T/E ratio of 0.24 shows that traffic is not the main cause of pollution here, since the levels of toluene are much lower than those of ethylbenzene. This is also shown by the B:T:E:X ratio of 3:0.24:1:1.9, with toluene being much less common. This could be because there are fewer emissions from traffic and more from natural or regional sources. Overall, these numbers show how much pollution is caused by traffic in PP compared to Snagov, which is cleaner and less affected by traffic.

**Figure 4:**
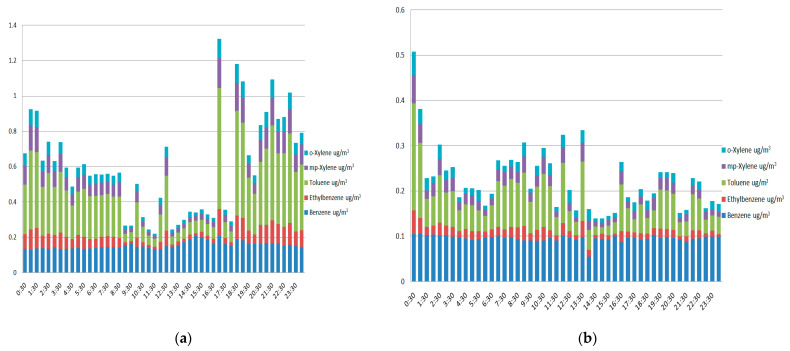
BTEX compounds during a sunny (**a**) and rainy day (**b**), measured in PP.

Figure 4 shows how BTEX changed throughout the day on both sunny and wet days. It showed two clear concentration peaks, one in the morning and one in the evening. Between 7:00 and 10:00 in the morning, the levels were at their highest. They then started to drop around 15:00. After that, from 17:00 until 22:00, when the second peak happened, BTEX levels rose again. This trend is a lot like the daily changes that can be seen in other cities.

The difference in BTEX compound amounts between the sunny day (25 August 2021) and the rainy day (26 October 2021) is due to weather conditions and traffic-related factors. On the sunny day, high levels of toluene, especially during rush hour, show how vehicle pollution affects the environment. Sunny days make it easier for BTEX compounds to build up because they slow down the mixing of the air, which lets pollution stay in the urban environment.

On the other hand, the overall BTEX concentrations are much lower on the rainy day, and there are no rush-hour peaks to be seen. In other words, it seems that wet weather spreads pollution and might also make traffic less heavy. On top of this, rain helps mix the air, which spreads emissions more evenly than on sunny days. Additionally, lower temperatures and less sunshine during rain can speed up chemical oxidation processes that may lower BTEX levels even more. This is especially true for compounds like toluene and xylene that react strongly with sunlight.

The fact that these trends show up again and again shows how weather affects pollution levels in cities. Even though traffic is a big source of BTEX pollution, the weather affects where these pollutants go and how they change chemically in the air. The data show that actions to lower traffic emissions might work best on sunny days. On the other hand, rain naturally helps reduce pollution, though only a small amount can be removed directly through wet deposition.

Table 4 shows how weather affects pollution trends by showing the difference in pollutant levels between sunny and cloudy/rainy days at the PP site. When it is warm outside, there are a lot more BTEX compounds in the air. This is probably because more cars are emitting them and the air is not moving as quickly, which stops pollutants from spreading. This effect is especially strong for toluene, which changes a lot when it is sunny, which suggests a strong link between traffic and the buildup of emissions when conditions are calm and the sun is out.

Nitrogen oxides (NO, NO_2_, and NOx) show similar patterns, with higher amounts on sunny days. This is likely because of more traffic and stronger photochemical reactions that make pollution build up. Also, ozone levels are much higher when it is sunny outside. This is because sunlight causes more formation, which makes it easier for photochemical processes to turn nitrogen oxides and VOCs into ozone.

BTEX and nitrogen oxide levels are much lower when it rains. This shows that rain helps to lower these pollutants, both by lowering traffic emissions (because people may drive less when it is raining) and by mixing and spreading the air more. These results show that rain indirectly lowers BTEX and NOx levels because the pollutants are spread out more effectively instead of being taken away directly by the rain.

It is interesting that while most emissions go down when it rains, carbon monoxide (CO) and sulfur dioxide (SO_2_) levels go up when it rains or is cold. This is probably because of more greenhouse gas releases and less spreading in the atmosphere when the air is colder and denser. The rise in CO and SO_2_ levels shows that heating systems in homes and businesses are a major source of these pollutants, especially when air flow is limited.

To sum up, most polluters, especially BTEX, nitrogen oxides, and ozone, are higher on sunny days because more pollution is released by cars and the weather is good for pollutant buildup. On the other hand, these pollutants tend to be lower on cold or rainy days. The only exceptions are CO and SO_2_, which are higher because of emissions tied to heating and less favorable conditions for spreading. These results show that weather and seasonal activities can change the patterns of pollution in cities. This shows how important it is to have customized methods for managing air quality that take these changes into account.

**Figure 5:**
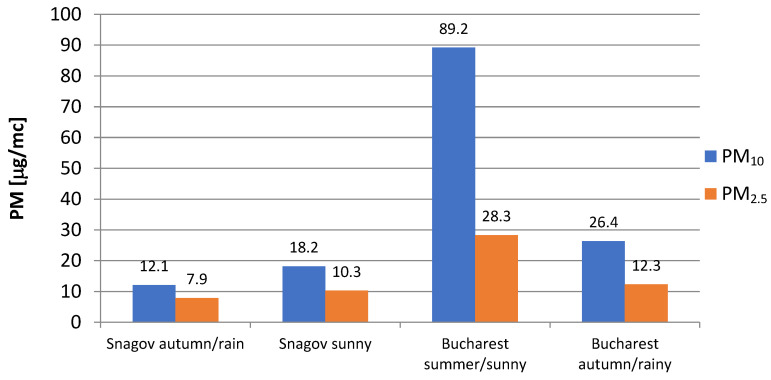
Particulate matter PM_2_._5_ and PM_10_ on a sunny and a rainy day.

Figure 5 shows data on PM_10_ and PM_2_._5_ amounts, which show how strongly weather and the number of people living in cities affect particulate matter levels. In Bucharest, PM levels are much higher in the summer and when it is sunny. This is because of pollution from cars, building sites, and factories, as well as still air that does not let pollutants move around. Particulate matter can build up in cities, especially when it is dry, because dry air makes it easier for particles to float around.

When it rains in Bucharest, PM levels drop by a lot. This drop is probably due to rain, which makes wet accumulation easier and helps remove particles from the air. However, even when it rains, the amount of particulate matter in the air in Bucharest is still higher than in rural places because of the constant pollution from cities.

In Snagov, a rural place with fewer sources of pollution, PM levels are always lower, no matter what the weather is like. This means that the air quality is better there. The small rise in particulate amounts on sunny days could be because there are not as many activities going on nearby or because particles are being moved farther away. However, these levels are still much lower than what was seen in Bucharest. Because Snagov is so different from Bucharest, this comparison shows how human activities raise particulate matter levels in cities, while rural areas benefit from natural settings that keep pollution levels low.

Overall, the data show that emissions and weather have a big effect on PM pollution in cities. Rainy weather helps lower particulate matter levels. But the difference in PM levels between cities and rural areas is still very big. This shows how important it is to focus pollution control in cities to deal with high amounts, especially when the weather is dry and still.

**Figure 6:**
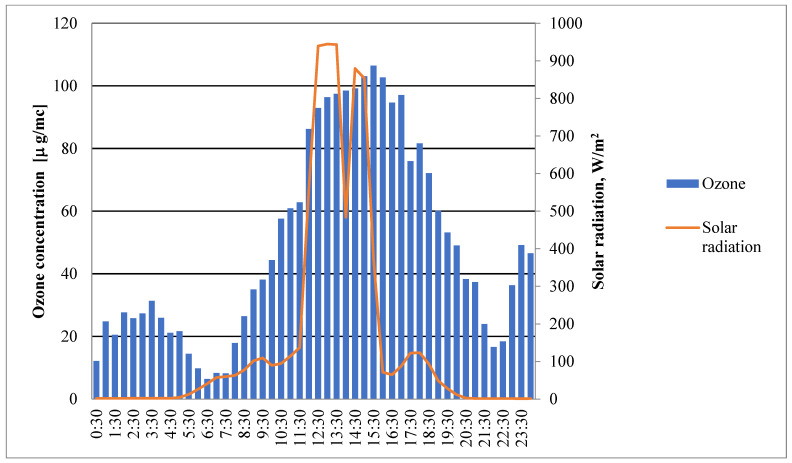
Concentrations of ozone on a sunny day in PP.

The data from the PP site on a sunny summer day (25 August 2021) illustrate the strong influence of solar radiation on ozone formation, as shown in Figure 6.

Low levels of solar energy and low levels of ozone in the early morning mean that there is not much photochemical activity. Ozone levels rise rapidly in the morning as sunlight strikes more strongly, causing photochemical reactions between sunlight and ozone precursors, mostly nitrogen oxides (NOx) and volatile organic compounds (VOCs).

Peak solar radiation causes ozone levels to be strongest in the middle of the day. This shows how important strong sunlight is for making ozone. In the afternoon, when solar radiation starts to decrease, ozone levels stay high. This suggests that ozone stays in the atmosphere after it is made because it is stable and precursor compounds are always present. This lasts into the late afternoon and early evening, which shows that ozone is still active in the atmosphere after peak sunlight hours, with amounts dropping slowly as sunlight decreases.

Overall, this trend shows how important solar radiation is for making ozone and how ozone levels drop slowly after peak sunlight. This study shows how important it is to control ozone precursors, especially in cities during the summer when strong sunlight can keep ozone levels high, causing health risks even as night falls.

**Figure 7:**
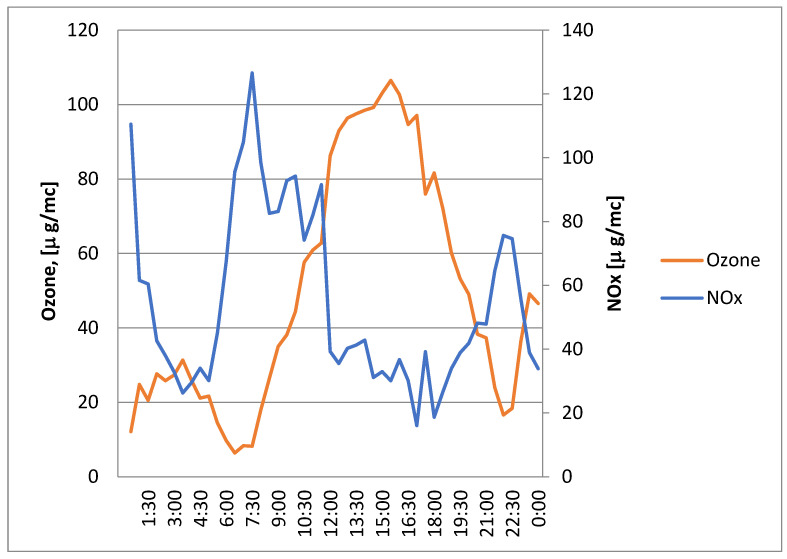
Concentrations of ozone compared to NOx concentrations.

The inverse link between NOx (nitrogen oxides) and ozone concentrations seen during the day, as shown in Figure 7, shows how photochemical processes work in city air. NOx levels are high at night and early in the morning because of constant emissions from cars and factories. On the other hand, ozone levels are low because photochemical ozone production is stopped by the lack of sunshine.

When it gets light outside, the sun’s rays start photochemical reactions with NOx and volatile organic compounds (VOCs), which make ozone. This process breaks down NOx, which lowers its levels while raising ozone levels. The highest levels of ozone are found in the afternoon, when the sun is shining the brightest and speeding up these photochemical processes.

As the day goes on, less sunshine slows down the process of ozone formation in the late afternoon and evening. Ozone levels start to drop, and NOx levels start to rise again. This is because emissions are still happening, but photochemical intake is going down. At night, NOx levels are high and ozone levels are low. During the day, NOx levels drop and ozone levels rise. This diurnal trend shows how emissions, chemical reactions, and weather conditions are constantly changing.

For managing air quality in cities, it is important to understand this inverse connection. It stresses that tactics meant to lower NOx emissions can have complicated impacts on the creation of ozone because their chemistry is linked. To effectively reduce pollution, we need a complete plan that takes into account when emissions happen, how sunlight affects the environment, and the presence of VOCs to deal with both main pollutants and secondary pollutants like ozone.

Table 5 delineates the regulation air quality thresholds established by the EU Directive to safeguard public health and the environment. It encompasses both limit values, denoting the maximum permissible concentrations of contaminants, and alert thresholds, signifying levels at which prompt intervention is required owing to health hazards. The enumerated pollutants—SO_2_, NO_2_, CO, ozone, and particle matter—serve as critical indicators of urban and industrial pollution, and their regulation is vital for upholding acceptable air quality requirements.

Table 6 presents the air quality standards established by the U.S. EPA, which function as legal thresholds for air pollutant concentrations aimed at safeguarding public health and the environment. The standards delineate exposure durations (1 h, 8 h, daily, and annual) to address both acute and chronic health risks. The table illustrates the stricter regulatory framework in the U.S. relative to certain other regions, particularly concerning pollutants such as SO_2_, NO_2_, CO, and particulate matter, indicating a significant focus on reducing exposure levels.

Table 7 provides an overview of the meteorological conditions at various monitoring sites, essential for analyzing pollutant dispersion and atmospheric behavior. Wind speed, temperature, pressure, and humidity are significant factors that influence pollutant concentrations and transport. These data contextualize air quality measurements, as weather conditions can exacerbate or mitigate pollution levels based on atmospheric stability and movement.

## 4. Conclusions

This study illustrates the efficacy of a mobile laboratory in delivering comprehensive, real-time pollution monitoring data in urban and rural settings, emphasizing the significant influence of traffic and weather conditions on air quality. The findings indicate that air quality in Bucharest, especially in high-traffic zones, is considerably poorer than in adjacent rural areas such as Snagov, characterized by increased concentrations of BTEX compounds, particulate matter, and nitrogen oxides. These pollutants are primarily associated with traffic emissions, highlighting the impact of vehicular pollution on urban air quality degradation.

Meteorological factors significantly influence pollution dynamics. During sunny days, elevated concentrations of pollutants such as BTEX, NOx, ozone, and particulate matter are noted, resulting from the accumulation of emissions in stable atmospheric conditions and enhanced photochemical activity facilitated by sunlight. Conversely, rainy days result in decreased pollution levels due to improved dispersion and chemical oxidation processes. However, pollutants such as CO and SO_2_ may rise on colder, rainy days due to emissions from heating and diminished dispersion. These observations underscore the complexity of pollutant behavior under different weather conditions, highlighting the necessity for adaptive air quality management strategies that account for seasonal and meteorological variations.

This research emphasizes the distinct pollution profiles observed in urban versus rural areas. Vehicular emissions are predominant in urban areas such as Bucharest, whereas rural locations like Snagov exhibit reduced pollution levels due to a scarcity of emission sources and natural mechanisms that facilitate pollutant dispersion. The BTEX ratios examined in this study highlight the significant impact of traffic in urban regions, while Snagov’s profile indicates a combination of localized or natural sources with limited influence from vehicular activity.

Rain significantly impacts traffic patterns, leading to reductions in traffic volume and operating speeds, particularly during adverse weather conditions. Studies consistently show that rainfall affects driver behavior, with more noticeable impacts on secondary roads compared to main roads.

This research provides important insights for policymakers, emphasizing the necessity of targeted pollution control measures in high-traffic urban areas. Effective strategies may encompass traffic reduction initiatives, the promotion of cleaner transportation options, and regulatory measures to limit emissions, especially during high-risk weather conditions such as sunny summer days. Future research should include diverse locations, such as industrial sites and educational facilities, to improve our understanding of air quality in various urban and rural contexts. This approach would establish a more comprehensive foundation for air quality management and public health protection.

This study highlights the significance of the continuous monitoring of pollutants and meteorological data for effective air quality management in urban settings.

## Figures and Tables

**Table 1:** A comparison of the measured BTEX values for other studies around the world.

Period	Area	Reference	City, Country	B µg/m^3^	T µg/m^3^	E µg/m^3^	X µg/m^3^	BTEX µg/m^3^	B:E [-]	T:E [-]	E:E [-]	X:E [-]
Summer 2015 and winter 2016	urban	[23]	Yazd, Iran	21.00	38.00	14.00	41.00	114.00	1.50	2.71	1.00	2.93
February2020 and September/October 2020	urban	[19]	Bandar Abbas, Iran	2.51	14.60	3.41	12.69	33.21	0.74	4.28	1.00	3.72
July to October 2013	urban	[14]	Rio de Janeiro, Brasil	1.50	6.70	1.50	4.20	13.90	1.00	4.47	1.00	2.80
May 2012 (summer)	urban	[13]	Dehradun, India	19.77	76.28	7.68	28.13	131.86	2.57	9.93	1.00	3.66
February 2013 (winter)				42.22	113.08	13.18	99.63	268.11	3.20	8.58	1.00	7.56
Spring, 2011	semi-urban (conversion with [26])	[24]	Orleans, France	1.61	0.59	0.08	0.20	2.48	19.54	7.16	1.00	2.48
Summer, 2011				0.37	0.56	0.09	0.23	1.25	4.25	6.36	1.00	2.58
Fall, 2010				0.36	1.58	0.16	0.49	2.59	2.17	9.67	1.00	3.01
Winter, 2010				0.94	1.23	0.21	0.67	3.05	4.51	5.88	1.00	3.21
Summer, 2004	urban	[29]	Beijing, China	13.40	16.10	5.40	14.60	49.50	2.48	2.98	1.00	2.70
Summer, 2005	urban	[29]	Paris, France	1.45	11.10	1.50	6.30	20.35	0.97	7.40	1.00	4.20
Summer, 2004	urban	[30]	Ramsis, Cairo, Egipt	87.20	213.80	43.30	214.57	558.87	2.01	4.94	1.00	4.96
	urban		Haram, Cairo, Egipt	46.23	111.80	22.77	110.85	291.65	2.03	4.91	1.00	4.87
	rural		Kafr El-Akram, Egipt	5.81	7.48	2.51	6.51	22.31	2.31	2.98	1.00	2.59
-	urban	[26]	Almaty, Kazakhstan	53.00	57.00	11.00	46.00	167.00	4.82	5.18	1.00	4.18
November 2017 to June 2018	urban	[12]	New Delhi, India	7.07	19.19	5.90	13.30	45.46	1.20	3.25	1.00	2.25
All year, 2016	urban	[31]	Arad, Romania	2.87	4.36	-	-	-	-	-	-	-
March 2012 to March 2013	urban	[32]	Tehran, Iran	3.44	3.63	16.25	17.26	40.59	0.21	0.22	1.00	1.06
August 2018, October 2018	urban	[33]	Leon, Mexico	1.96	12.92	13.19	3.51	31.58	0.15	0.98	1.00	0.27
January to December 2012	urban	[34]	Gdansk, Gdynia, and Sopot, Poland	0.66	1.07	0.28	0.85	2.85	2.38	3.87	1.00	3.09
March 2006 and February 2007	urban	[35]	Valencia, Spain	1.20	6.80	0.90	1.40	10.30	1.33	7.56	1.00	1.56
April, Septeber to October 2008	urban	[36]	Bari, Italy	1.50	1.44	1.28	1.29	5.51	1.18	1.13	1.00	1.01
September 2017 to early January 2018	urban	[37]	Klang Valley Region, Malaysia	25.82	89.08	23.89	73.04	211.83	1.08	3.73	1.00	3.06
April to July 2016	urban	[7]	Bucharest, Romania	0.97	2.36	2.37	4.32	10.02	0.41	0.99	1.00	1.82

**Table 2:** Descriptions of the equipment of the mobile lab.

Pollutant	Name	Measurement Principle	Measuring Range	Detection Limit	Accuracy %
BTEX	GC 5000 AMA	GC/FID	0–50 µg/m^3^	0.03 ppb	±1.0%
NOx	APNA 370 Horiba	Chemiluminescence	0–1.0 ppm	0.5 ppb	±1.0%
Ozone	APOA 370 Horiba	Ultraviolet absorption	0–1.0 ppm	0.5 ppb	±1.0%
CO	APMA 370 Horiba	Non-dispersive infrared absorption	0–100 ppm	0.05 ppm	±1.0%
SO_2_	APSA 370 Horiba	Ultraviolet fluorescence	0–0.5 ppm	0.5 ppb	±1.0%
PM_10_	F701-20 Verewa	Beta-radiation adsorption	0–10 mg/m^3^	0.001 mg/m^3^	±2.0%
PM_2.5_	Dust Monitor 11-E	Laser radiation	0.25–32 µm	0.1 µg/m^3^	±2.0%

**Table 3:** Ratios of B/T, T/B, B/E, T/E, m, p X/E, and B ÷ T ÷ E ÷ X at two analyzed sites.

Site	B/T	T/B	B/E	T/E	m, p X/E	B ÷ T ÷ E ÷ X
PP	0.62	1.62	2.38	3.86	1.20	2.3:3.8:1:2
Snagov	0.73	1.38	3.01	0.24	1.15	3:0.24:1:1.9

**Table 4:** Comparison of emissions, presented as average ± SD, on a sunny day and a rainy day at the site PP, µg m^−3^.

Pollutant	Sunny Day	Cold/Rainy Day
Benzene	0.156 ± 0.02	0.096 ± 0.007
Toluene	0.252 ± 0.166	0.07 ± 0.043
Ethylbenzene	0.065 ± 0.039	0.019 ± 0.09
m,p-Xylene	0.078 ± 0.044	0.023 ± 0.01
o-Xylene	0.053 ± 0.028	0.018 ± 0.009
NO	7.673 ± 10.143	6.683 ± 12.535
NO_2_	42.857 ± 16.075	25.418 ± 13.922
NOx	54.579 ± 27.385	35.634 ± 30.978
Ozone	42.218 ± 32.136	27.023 ± 15.071
CO *	0.259 ± 0.075	0.440 ± 0.0.134
SO_2_	1.226 ± 0.690	3.819 ± 0.255

* mg/m^3^.

**Table 5:** Value limits and alert thresholds, according to Directive 50/2008/EC.

Pollutant	Limit Values				Alert Thresholds
	1 h	8 h	1 day	Annual	
SO_2_, µg/m^3^	350		125		500
NO_2_, µg/m^3^	200			40	400
CO, mg/m^3^		10			
Ozone, µg/m^3^		120			240
Benzene, µg/m^3^				5	
PM_10_, µg/m^3^			50	40	
PM_2_._5_, µg/m^3^				24	

**Table 6:** Romanian Ambient Air Quality Standards—EPA, 2013.

Pollutant	Limit Values				
	1 h	3 h	8 h	1 day	Annual
SO_2_	75 ppbv (196 µg/m^3^)	0.5 ppmv (1300 µg/m^3^)		0.14 ppmv (365 µg/m^3^)	0.03 ppmv (80 µg/m^3^)
NO_2_	100 ppbv (188 µg/m^3^)				53 ppbv (100 µg/m^3^)
CO	35 ppmv (40 mg/m^3^)		9 ppmv (10 mg/m^3^)		
Ozone	0.12 ppmv (235 µg/m^3^)		0.075 ppmv (147 µg/m^3^)		
PM_10_				150 µg/m^3^	50 µg/m^3^
PM_2.5_				35 µg/m^3^	15 µg/m^3^

**Table 7:** Measured meteorological data.

Site *	Wind Speed m/s	Temperature °C	Sea Level Pressure mbar	Relative Humidity %
1	1.2 ± 0.6	33.2 ± 4.2	1008 ± 2.8	50 ± 1
2	1.4 ± 1.5	16.3 ± 5.7	1003 ± 4.9	55 ± 3

* PP -1; S -2.

## Data Availability

Data are contained within the article.
